# Supplementary material for: Selecting Optimal Proton Pencil Beam Scanning Plan Parameters to Reduce Dose Discrepancy between Discrete Spot Plan and Continuous Scanning: A Proof-of-Concept Study
Source: Cancers (Basel). 2023 Aug 13;15(16):4084. doi: 10.3390/cancers15164084 (PMC10452710; doi:10.3390/cancers15164084)
Supplement: Supplementary file 1 [file cancers-15-04084-s001.zip › cancers-2441718-supplementary.pdf]

cancers

MDPI

Supplementary Materials

Selecting Optimal Proton Pencil Beam Scanning Plan Parameters to Reduce Dose Discrepancy between Discrete Spot Plan and Continuous Scanning: A Proof-of-Concept Study

Xiaoying Liang, Chris J. Beltran, Chunbo Liu, Chunjoo Park, Bo Lu, Sridhar Yaddanapudi, Jun Tan and Keith M. Furutani

Table S1. Simulated DDCS Dose Discrepancy and Fraction of Break Spots.

MU/spot minimum, mMU	Spot spacing, sigma	Dose, GyRBE	Stop ratio constraint	Break spots fraction, %	CTV DVH RMSE, %	2%/2-mm gamma passing rate, %	1%/1-mm gamma passing rate, %
0.1	0.5	0.5	1	90	0.0	100.0	100.0
0.1	1.0	0.5	1	78	0.3	98.0	91.1
0.1	1.5	0.5	1	71	0.2	98.5	90.9
3.0	0.5	0.5	1	49	0.6	98.2	89.9
3.0	1.0	0.5	1	39	1.0	98.4	87.8
3.0	1.5	0.5	1	49	0.3	99.6	96.7
6.0	1.0	0.5	1	25	1.9	93.3	78.8
0.1	0.5	2.0	1	63	0.0	100.0	100.0
0.1	1.0	2.0	1	43	0.1	100.0	99.4
0.1	1.5	2.0	1	25	0.1	100.0	100.0
3.0	0.5	2.0	1	29	0.1	100.0	100.0
3.0	1.0	2.0	1	23	0.1	100.0	100.0
3.0	1.5	2.0	1	14	0.0	100.0	100.0
6.0	0.5	2.0	1	11	0.9	99.4	91.3
6.0	1.0	2.0	1	8	0.4	100.0	97.7
6.0	1.5	2.0	1	6	0.1	100.0	99.9
0.1	0.5	0.5	0	75	0.0	100.0	100.0
0.1	1.0	0.5	0	58	0.6	98.0	91.1
0.1	1.5	0.5	0	40	0.3	98.5	90.9
3.0	0.5	0.5	0	22	2.5	86.5	74.2
3.0	1.0	0.5	0	16	1.6	95.2	82.9
3.0	1.5	0.5	0	21	1.0	96.3	84.9
6.0	1.0	0.5	0	7	4.5	81.4	63.5
0.1	0.5	2.0	0	39	0.0	100.0	100.0
0.1	1.0	2.0	0	29	0.1	100.0	99.7
0.1	1.5	2.0	0	19	0.1	100.0	99.8
3.0	0.5	2.0	0	9	0.2	100.0	99.9
3.0	1.0	2.0	0	8	0.2	100.0	99.8
3.0	1.5	2.0	0	5	0.1	100.0	100.0
6.0	0.5	2.0	0	3	3.7	79.3	65.4
6.0	1.0	2.0	0	3	1.1	97.0	87.9
6.0	1.5	2.0	0	0	0.2	100.0	99.4
3.0	0.5	5.0	0	2	0.1	100.0	100.0

3.0	1.0	5.0	0	2	0.1	100.0	100.0
3.0	1.5	5.0	0	2	0.0	100.0	100.0
6.0	0.5	5.0	0	1	0.2	97.9	80.6
6.0	1.0	5.0	0	0	0.1	100.0	100.0
6.0	1.5	5.0	0	1	0.0	100.0	100.0
3.0	0.5	10.0	0	0	0.1	100.0	100.0
3.0	1.0	10.0	0	1	0.1	100.0	100.0
3.0	1.5	10.0	0	2	0.0	100.0	100.0
6.0	0.5	10.0	0	0	0.2	100.0	100.0
6.0	1.0	10.0	0	0	0.1	100.0	100.0
6.0	1.5	10.0	0	0	0.0	100.0	100.0

Abbreviations: CTV, clinical target volume; DDCS, dose-driven continuous scanning; DVH, dose-volume histogram; GyRBE, relative biological effectiveness of physical dose in Gray; MU, monitor unit; RMSE, root mean square error.
